# Capsular Serotype and Antibiotic Resistance of *Streptococcus pneumoniae* Isolates in Malaysia

**DOI:** 10.1371/journal.pone.0019547

**Published:** 2011-05-16

**Authors:** Cheng-Foh Le, Navindra Kumari Palanisamy, Mohd Yasim Mohd Yusof, Shamala Devi Sekaran

**Affiliations:** 1 Department of Medical Microbiology, Faculty of Medicine, University of Malaya, Kuala Lumpur, Malaysia; 2 Faculty of Medicine, Universiti Teknologi MARA, Shah Alam, Selangor; Universita di Sassari, Italy

## Abstract

**Background:**

*Streptococcus pneumoniae* is a major causative agent of severe infections, including sepsis, pneumonia, meningitis, and otitis media, that has since become a major public health concern. In this study, the serotypes distribution of pneumococcal isolates was investigated to predict the efficacy of the 7-valent pneumococcal conjugate vaccine (PCV7) among the Malaysian populations.

**Methodology/Principal Findings:**

A total of 151 clinical isolates were serotyped using multiplex PCR assays. Out of them, there were 21.2% penicillin-resistant, 29.1% penicillin-intermediate, and 49.7% penicillin-susceptible *S.pneumoniae* strains. Serotypes detected among the Malaysian isolates were 1, 3, 10A, 11A/11D, 12F/12A, 14, 15A, 15B/15C, 16F, 18C/18B/18A/18F, 19A, 19F, 23F, 35B, 35F/47F, 6A/6B, 7C/7B/40, 7F/7A, 9V/9A, and 34. Serotype 19F and 23F were the two most prevalent serotypes detected. Serotypes are highly associated with invasiveness of isolates (p = 0.001) and penicillin susceptibility (p<0.001). Serotype 19F was observed to have increased resistance against penicillin while serotype 19A has high invasive tendency. Age of patients was an important factor underlying the pneumococcal serotypes (p = 0.03) and clinical sites of infections (p<0.001). High prevalence of pneumococcal isolates were detected among children <5 years old at nasopharyngeal sites while elderly adults ≥60 years old were at increased risk for pneumococcal bacteremia.

**Conclusion/Significance:**

Current study revealed that a number of serotypes, especially those associated with high penicillin resistance, have been formulated in the PCV7. Therefore, the protections expected from the routine use of PCV7 would be encouraging for the Malaysian. However, it is not possible to predict serotypes that might become predominant in the future and hence continued surveillance of circulating serotypes will be needed.

## Introduction

*Streptococcus pneumoniae* is a major causative agent of the morbidity and mortality among young children and adults [1]. More than one million children die of pneumococcal diseases worldwide each year and the incidences are especially high in developing countries. In contrast to the developed countries where pneumococcal respiratory infections predominantly affected the elderly group, death in children under 5 years of age in the developing world are attributed to pneumococcal diseases. Among them, children under 2 years old are typically the most severely affected group.

An important contributing factor to the invasiveness and hence the ability of the organism to cause diseases is the production of polysaccharide capsule. Pneumococci are able to produce at least 90 immunologically distinct capsules that differ in their chemical properties [2]. The capsule which present external to the cell wall of pneumococcus disrupts the neutrophil-mediated and complement activity of bacterial cells leading to inhibition of opsonophagocytosis [3]. This confers the organism with resistance against phagocytosis and promotes bacterial evasion from the host immune system [4]. Of the 90 capsular types, only a few are causative agents of invasive diseases and associated with pediatric diseases [5]. Serotypes 4 and 14 and serogroups 6, 7, 9, 18, 19, and 23 are known to be associated with pediatric diseases [5]. However, the association between serotypes/serogroups and diseases incidences varied depending upon the geographical distributions and time period of the investigations [5].

The prevalence and distributions of *S.pneumoniae* in Malaysia are closely linked to neighboring countries within South East Asia region and other countries in Asia [6]. In Malaysia, several research groups have conducted investigations on the serotype distributions in our population. This provides some clues though still inadequate to give a comprehensive presentation of pneumococcal diseases. A number of surveillance studies revealed that pneumonia is the most common clinical presentation of pneumococcal infection, with those children <2 years of age accounted for the most morbidity and mortality cases [7]–[9]. Other invasive pneumococcal diseases (IPD) among local populations are meningitis, septic arthritis, and bacteremia without focus (BWF) [9]. Studies on serotypes distributions in Malaysia revealed differing outcomes probably due to the lack of consistent and continuous monitoring of the serotypes in the same populations, time interval between study, scale, and the geographical dissemination of the study. A four years study conducted at a major referral hospital in Kuala Lumpur identified the predominant serotypes to be 6A, 6B, 14, and 19A [7]. In a separate study by Rohani et al., they found serotypes 1, 2, 5, 6A, 8, 12A, 14, 19B, 20, and 23B were the most common serotypes implicated in IPD [8]. However, whether these revealed a shift in serotypes prevalence is unclear as the data could not be well correlated to draw a definite conclusion.

Since the emergence of penicillin resistant strain in 1967, antibiotic resistance strains of *S. pneumoniae* have spread worldwide. The organism has also been reported to be resistant to other antibiotics such as macrolides and quinolones [10]–[11]. Despite the availability of multiple conventional antibiotics as well as the new generation broad spectrum antibiotics with improved efficacy, the management of pneumococcal infections has become difficult with the rapid development of antimicrobial resistance in this organism. Although the beta-lactam antibiotics, particularly penicillin still remains as the main choice in antibiotic therapy against pneumococcal diseases, the alarming rise of penicillin resistance as well as others has prompted for the reconsideration of better control and prevention strategies.

The majority of antibiotic resistance strains carry serotypes which are not included in the currently in use conjugate vaccines [12]. It has been reported that serotypes reported in both pediatric and adult infections are frequently found among both drug susceptible and resistant strains that colonize healthy children [13]. Therefore, the main focus of better management of pneumococcal infection is currently through widespread usage of vaccination. The available pneumococcal polysaccharide vaccine (PPV) covers 23 serotypes, which represents 90% of the strains responsible for invasive diseases but are found to be not immunogenic in young children. The alternative to this is the heptavalent pneumococcal conjugate vaccine (PCV7) which is immunogenic in children <2 years old. Apart from PCV7, PCV with additional formulations depending on the manufacturers are also available. This includes the 7-, 9-, 10-, and 13-valent PCV [14] that confer protection against different number of pneumococcal serotypes.

All these clearly outlined the impact of pneumococcal diseases to the populations especially for the young children. However, this demanding information is still scarce despite the rising threats of pneumococcal infections have been persisted among the Malaysian populations for many years. As carriages usually precede pneumococcal diseases and the natures of invasiveness are correlated to different pneumococcal serotypes, data on serotypes distribution among local populations are needed. Hence, to evaluate the coverage and the extent of potential protection conferred by PCV7, the serotypes specific epidemiology distributions of pneumococcus from the University Malaya Medical Centre (UMMC) was investigated.

## Materials and Methods

### Clinical isolates collection and culture conditions

A total of 151 pneumococcal isolates were obtained from clinical samples processed stored at the Microbiology Laboratory of the University of Malaya Medical Centre, Malaysia from March 1999 to February 2007 (except year 2001 and 2004). Control strains of known serotypes (Quellung reaction) representing different serotypes and serogroups were used in the study. The serotypes included 1, 2, 3, 4, 5, 8, 13, 14, 20, 21, 31, 34, 37, 38, 39, 40, 44, 46, 6A, 7 A, 7B, 7C, 7F, 9A, 9N, 9L, 9L, 10A, 10F, 11 A, 11D, 11F, 12A, 12B, 12F, ISA, 15B, 15C, 15F, 16A, 16F, 17F, 33A, 35B, 35F, 35A, 35C, and 47F. The isolates were obtained from invasive and non invasive sites of both pediatric and adult patients. The source of the isolates included blood, cerebrospinal fluid (CSF), nasopharyngeal (NP) secretion, tracheal secretion, sputum, bronchoalveolar lavage (BAL), and others. Samples were grown on 5% horse blood agar and incubated at 37°C in the presence of 5% CO_2_ for 12–15 hours prior to other biochemical and molecular assays.

### Ethics Statement

The Ethical Committee of the University of Malaya Medical Centre has exempted this study from review as these isolates were from routine diagnosis and waived the need for consent due to the fact that neither patient information nor identity was being used, related, or involved in any aspect of the study.

### Isolates identification

The isolates were identified as *S. pneumoniae* using conventional microbiological methods including susceptibility to ethylhydrocupreine disc (optochin), catalase test and bile solubility.

### Susceptibility testing

The antibiotic susceptibility of the strains was tested on Mueller Hinton Agar (Oxoid) plates containing 5% sheep blood (Oxoid), incubated at 37°C with 5% CO_2_ using the agar dilution method as described [15]. The penicillin used was obtained from Sigma Aldrich (Sigma Chemical Co., St. Louis, Mo). *S. pneumoniae* ATCC 49619 was used as control.

### DNA extraction

Genomic DNA was extracted from the bacterial culture using a previously described method [16]. Bacterial colonies suspended in 15 µl of dH_2_O were treated with 10 µg/ml Proteinase K and 0.lmM Tris HCL pH 7.5 and incubated at 37°C for another 10 minutes. Subsequently, the suspension was boiled for 5 minutes and finally centrifuged at 13000 rpm for 2 minutes. The supernatant obtained was used as the template in the PCR reaction.

### PCR amplification

**Figure 1:**
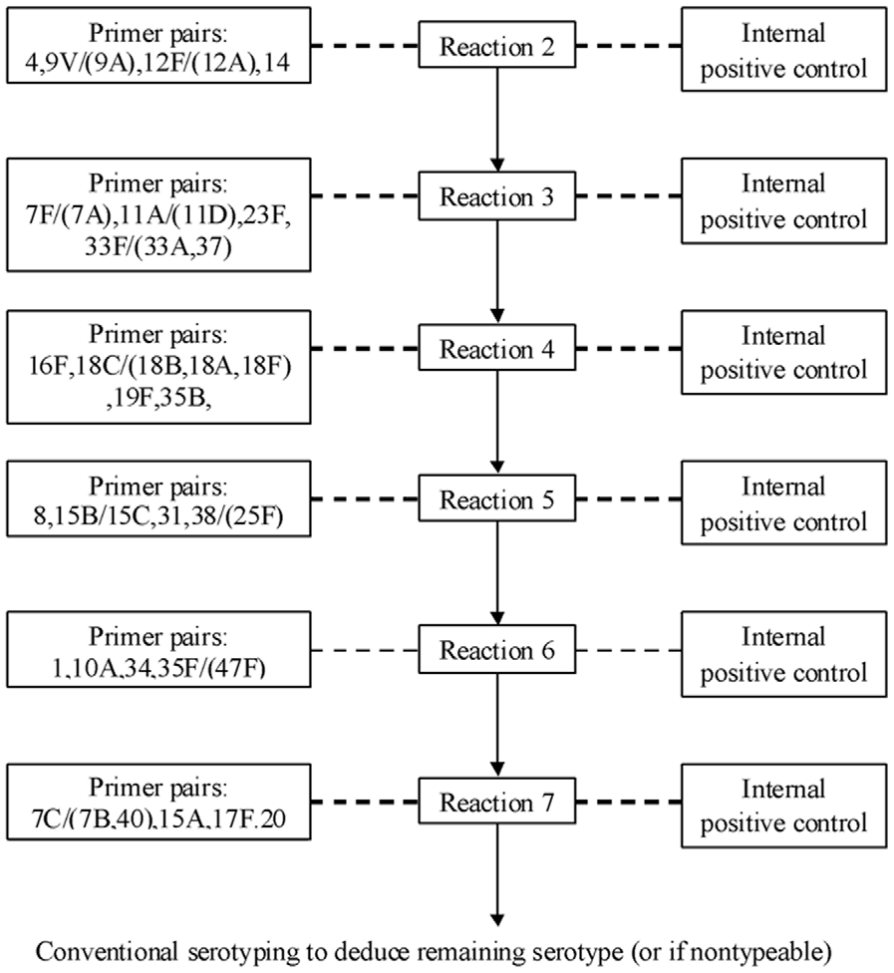
Sequential multiplex PCR approaches for pneumococcal capsular serotyping. Each of the seven reactions include five primer pairs, four of which targets four different serotypes and another primer pair serves as internal positive control.

The primers used in this study were extracted from previously published sequences and the PCR protocol was as previously described [17]. Briefly, the primers were grouped into seven multiplex reactions as shown in Figure 1 and each reaction was designed to include four primer pairs targeting four different serotypes and another primer pair targeting the common region of the cps operon as an internal positive control. The optimal PCR condition for a 25 µ1 reaction included 1× PCR buffer (Fermentas, Lithuania), 2.5 mM MgCl_2_, 0.2 mM dNTP mix, 2 U Taq Polymerase (Fermentas) and primer pairs at varying concentrations as shown in Table S1 [17]. The PCR cycling was carried out in an Eppendorf Gradient Mastercycler with initial denaturation step at 94°C for 4 minutes and the 30 amplification cycles were performed with denaturation at 94°C for 45 s, annealing temperature at 54°C for 45 s and extension temperature at 65°C for 2 minutes and 30 s and finally completed with an extension at 72°C for 2 minutes. The PCR product was electrophoresed on a 2% TAB agarose gel for 1 hour at 70V and the bands were analyzed using a UV transilluminator.

### Statistical analysis

Association among serotypes, clinical sites of isolates, age groups, and penicillin susceptibility were tested using Chi-square test of independence or Fisher's exact test, whenever appropriate.

## Results

### Minimal inhibitory concentrations

The isolates were categorized based on their susceptibility to penicillin, termed as penicillin-susceptible *S. pneumoniae* (S_p_), penicillin-intermediate *S. pneumoniae* (I_p_), and penicillin-resistant *S. pneumoniae* (R_p_). Penicillin-intermediate and -resistant strains were collectively referred to as penicillin nonsusceptible strains. Of the 151 isolates, there were 21.2% (32/151) R_p_, 29.1% (44/151) I_p_, and 49.7% (75/151) S_p_ (Table 1). The prevalence of penicillin susceptible to nonsusceptible strains was at near equal ratio (penicillin susceptible 49.7% to penicillin nonsusceptible 50.3%).

**Table 1:** Pneumococcal serotypes distribution with respect to penicillin susceptibility.

	Frequency (adjusted residual, % within serotype, % within susceptibility group)													
Serotype^c^	Susceptible				Intermediate				Resistant				Total^a^	
1	7	(1.7,	77.8,	9.3)	2	(−0.5,	22.2,	4.5)	0	(−1.6,	0.0,	0.0)	9	(6.0)
3	1	(1.0,	100.0,	1.3)	0	(0.6,	0.0,	0.0)	0	(−0.5,	0.0,	0.0)	1	(0.7)
14	1	(−1.3,	20.0,	1.3)	4	(2.5,	80.0,	9.1)	0	(−1.2,	0.0,	0.0)	5	(3.3)
34	0	(−1.4,	0.0,	0.0)	0	(−0.9,	0.0,	0.0)	2	(2.7,	100.0,	6.3)	2	(1.3)
10A	1	(1.0,	100.0,	1.3)	0	(−0.6,	0.0,	0.0)	0	(−0.5,	0.0,	0.0)	1	(0.7)
11A/11D	1	(−0.6,	33.3,	1.3)	2	(1.4,	66.7,	4.5)	0	(−0.9,	0.0,	0.0)	3	(2.0)
12F/12A	3	(1.8,	100.0,	4.0)	0	(−1.1,	0.0,	0.0)	0	(−0.9,	0.0,	0.0)	3	(2.0)
15A	1	(1.0,	100.0,	1.3)	0	(−0.6,	0.0,	0.0)	0	(−0.5,	0.0,	0.0)	1	(0.7)
15B/15C	1	(1.0,	100.0,	1.3)	0	(−0.6,	0.0,	0.0)	0	(−0.5,	0.0,	0.0)	1	(0.7)
16F	2	(1.4,	100.0,	2.7)	0	(−0.9,	0.0,	0.0)	0	(−0.7,	0.0,	0.0)	2	(1.3)
18C	2	(1.4,	100.0,	2.7)	0	(−0.9,	0.0,	0.0)	0	(−0.7,	0.0,	0.0)	2	(1.3)
19A	7	(2.2,	87.5,	9.3)	1	(−1.1,	12.5,	2.3)	0	(−1.5,	0.0,	0.0)	8	(5.3)
19F	12	(−5.3,	21.4,	16.0)	24	(2.8,	42.9,	54.5)	20	(3.4,	35.7,	62.5)	56	(37.1)
23F	9	(0.6,	56.3,	12.0)	1	(−2.1,	6.3,	6.3)	6	(1.7,	37.5,	18.8)	16	(10.6)
35B	2	(0.0,	50.0,	2.7)	2	(0.9,	50.0,	4.5)	0	(−1.1,	0.0,	0.0)	4	(2.6)
35F/47F	2	(0.6,	66.7,	2.7)	1	(0.2,	33.3,	2.3)	0	(−0.9,	0.0,	0.0)	3	(2.0)
6A/6B	3	(−1.0,	33.3,	4.0)	5	(1.8,	55.6,	11.4)	1	(−0.8,	11.1,	3.1)	9	(6.0)
7C/7B/40	1	(1.0,	100.0,	1.3)	0	(−0.6,	0.0,	0.0)	0	(−0.5,	0.0,	0.0)	1	(0.7)
7F/7A	3	(1.8,	100.0,	4.0)	0	(−1.1,	0.0,	0.0)	0	(−0.9,	0.0,	0.0)	3	(2.0)
9V/9A	0	(−1.0,	0.0,	0.0)	0	(−0.6,	0.0,	0.0)	1	(1.9,	100.0,	3.1)	1	(0.7)
ND	16	(2.9,	80.0,	21.3)	2	(−2.0,	10.0,	4.5)	2	(−1.3,	10.0	6.3)	20	(13.2)
Total^b^	75 (49.7)				44 (29.1)				32 (21.2)				151	

a Frequency (total % within serotype).

b Frequency (total % within susceptibility groups).

c Fisher's exact test, p<0.001.

Abbreviation: ND, not-detected.

### Serotyping

**Figure 2:**
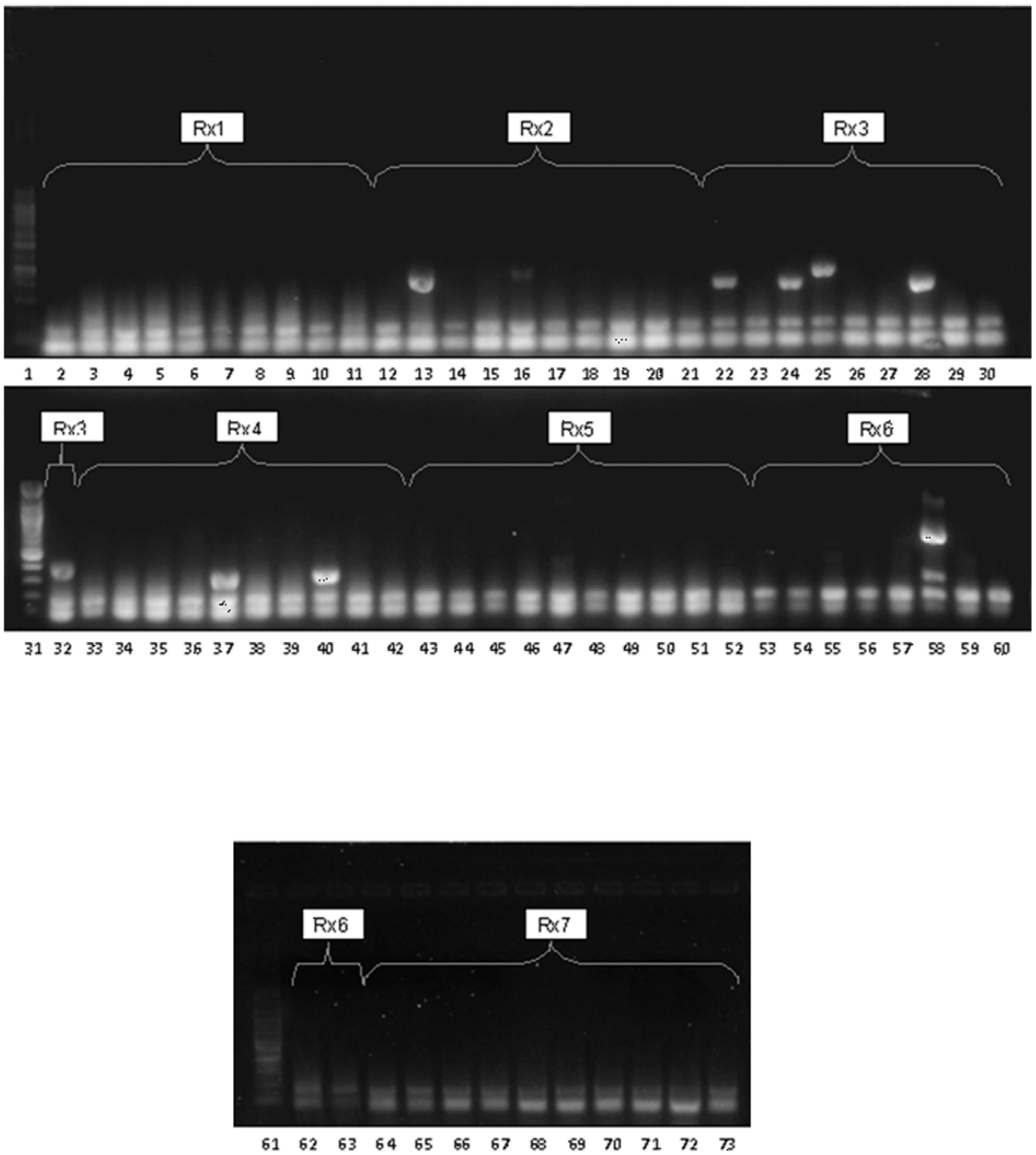
Representative complete seven multiplex reactions performed on 10 R_p_ strains for serotyping. In each reaction, the strains were arranged in the same position accordingly from left to right. The serotypes detected were: PR40(1), PR41(12F), PR44(19F), PR46(11A/D), PR47(12F), PR48(1), PR49(23F), PR50(19F), PR55(not-detected), and PR56(23F) (annotations in parenthesis denotes the serotype; ND: not-detected). Lane 1, 31, 61: 100 kb DNA ladder.

Serotypes detected among the Malaysian isolates were 1, 3, 10A, 11A/11D, 12F/12A, 14, 15A, 15B/15C, 16F, 18C/18B/18A/18F, 19A, 19F, 23F, 35B, 35F/47F, 6A/6B, 7C/7B/40, 7F/7A, 9V/9A, and 34. Figure 2 shows a representative complete seven multiplex reactions performed on 10 R_p_ strains. Serotype 19F was the most prevalent serotype (37.1%, 56/151) followed by serotype 23F (10.6%, 16/151), serotype 1 (6.0%, 9/151) and serotype 6A/6B (6.0%, 9/151) irrespective of their susceptibility to penicillin. Together, serotype 19F and 23F accounted for almost half of all cases (47.7%). As the largest serotype group, 78.6% (44/56) of serotype 19F were nonsusceptible to penicillin (Table 1). Out of them, 35.7% were penicillin-resistant. Besides this, a pool of uncommon serotypes were detected including serotype 3, 10A, 12F/12A, 15A, 15B/15C, 16F, 18C/18B/18A/18F, 7C/7B/40, and 7F/7A. Statistical analysis showed significant relationship between serotype and penicillin susceptibility (p<0.001). In particular, serotype 19F was the most significantly associated group against penicillin resistance.

Majority of the clinical isolates were obtained from non invasive sites (72.2%, 109/151). Among the non invasive sites, sputum (27.8%, 42/109) and NP (25.2%, 38/109) constituted the two main sites where pneumococcal isolates have been frequently isolated (Table 2). Serotype 19F was determined to be the dominant serotype in these two clinical sites (NP = 40.0%, sputum = 32.0%). In addition, sputum, NP, and blood represent the three major sites of pneumococcal isolations which accounted for 77.5% (117/151) of all isolates. Other clinical sites included tracheal secretion (7.9%), ear swab/pus (6.0%), pleural fluid (2.0%), eye swab/pus (2.0%), cerebrospinal fluid 1.3%), broncheoalveolar lavage (1.3%), and others (2.0%). Isolates from rare sites such as Bartholin's abscess and vaginal discharge were reported in this study as well. Serotypes were associated with invasiveness of the isolates (p = 0.001) where serotype 19A was associated with invasive sites (Table 3).

**Table 2:** Distribution of pneumococcal isolates based on age groups against the clinical sites of isolations.

	Invasive			Non invasive							
Age^c^	Blood	CSF	Pleural fluid	Sputum	NP	Eye swab/pus	Ear swab/pus	Tracheal secretion	BAL	Other^d^	Total^a^
<5	7	1	1	2	28	0	5	2	1	0	47(31.1)
5–59	16	1	2	27	10	3	4	6	0	2	71(47.0)
≥60	14	0	0	13	0	0	0	4	1	1	33(21.9)
Total^b^	37(24.5)	2(1.3)	3(2.0)	42(27.8)	38(25.2)	3(2.0)	9(6.0)	12(7.9)	2(1.3)	3(2.0)	151

a Frequency (total % within age groups).

b Frequency (total % within clinical sites).

c Fisher's exact test, p<0.001.

d Other sites include left tonsil, Bartholin's abscess, and vaginal discharge.

Abbrevations: CSF, cerebrospinal fluid; NP, nasopharyngeal; BAL, bronchoalveolar lavage.

**Table 3:** Pneumococcal serotypes distribution with respect to invasiveness of isolates.

	Frequency (adjusted residual, % within serotype, % within invasiveness)									
Serotype^c^	Invasive				Non invasive				Total^a^	
1	3	(0.4,	33.3,	7.1)	6	(−0.4,	66.7,	5.5)	9	(6.0)
3	0	(−0.6,	0.0,	0.0)	1	(0.6,	100.0,	0.9)	1	(0.7)
14	3	(1.6,	60.0,	7.1)	2	(−1.6,	40.0,	1.8)	5	(3.3)
34	0	(−0.9,	0.0,	0.0)	2	(0.9,	100.0,	1.8)	2	(1.3)
10A	0	(−0.6,	0.0,	0.0)	1	(0.6,	100.0,	0.9)	1	(0.7)
11A/11D	2	(1.5,	66.7,	4.8)	1	(−1.5,	33.3,	0.9)	3	(2.0)
12F/12A	2	(1.5,	66.7,	4.8)	1	(−1.5,	33.3,	0.9)	3	(2.0)
15A	0	(−0.6,	0.0,	0.0)	1	(0.6,	100.0,	0.9)	1	(0.7)
15B/15C	1	(1.6,	100.0,	2.4)	0	(−1.6,	0.0,	0.0)	1	(0.7)
16F	1	(0.7,	50.0,	2.4)	1	(−0.7,	50.0,	0.9)	2	(1.3)
18C	0	(−0.9,	0.0,	0.0)	2	(0.9,	100.0,	1.8)	2	(1.3)
19A	6	(3.1,	75.0,	14.3)	2	(−3.1,	25,0,	1.8)	8	(5.3)
19F	6	(−3.6,	10.7,	14.3)	50	(3.6,	89.3,	45.9)	56	(37.1)
23F	4	(−0.3,	25.0,	9.5)	12	(0.3,	75.0,	11.0)	16	(10.6)
35B	1	(−0.1,	25.0,	2.4)	3	(0.1,	75.0,	2.8)	4	(2.6)
35F/47F	1	(0.2,	33.3,	2.4)	2	(−0.2,	66.7,	1.8)	3	(2.0)
6A/6B	1	(−1.2,	11.1,	2.4)	8	(1.2,	88.9,	7.3)	9	(6.0)
7C/7B/40	1	(1.6,	100.0,	2.4)	0	(−1.6,	0.0,	0.0)	1	(0.7)
7F/7A	1	(0.2,	33.3,	2.4)	2	(−0.2,	66.7,	1.8)	3	(2.0)
9V/9A	1	(1.6,	100.0,	2.4)	0	(−1.6,	0.0,	0.0)	1	(0.7)
ND	8	(1.3,	40.0,	19.0)	12	(−1.3,	60.0,	11.0)	20	(13.2)
Total^b^	42 (27.8)				109 (72.2)				151	

a Frequency (total % within serotype).

b Frequency (total % within invasiveness).

c Fisher's exact test, p = 0.001.

Abbreviation: ND, not-detected.

Statistical testing showed significant relationship between the age of patients and serotypes (p = 0.03) (Table 4). Patients aged 5–59 years old represented the largest age group which accounted for approximately half of all cases (47.0%, 71/151). High proportions of serotype 19A (75.0%), 23F (75.0%), and 6A/6B (66.7%) were isolated from patients within this age range. In addition, clinical sites of pneumococcal isolations were highly associated with the age of patients (p<0.001) (Table 2) whereby pneumococci were frequently isolated from NP site of children <5 years old. As high as 80.9% (38/47) of pneumococcal isolates in children <5 years of age were sourced from non invasive sites particularly the NP sites (59.6%, 28/47). No association was observed between age of patients and penicillin susceptibility (p = 0.551).

**Table 4:** Pneumococcal serotypes distribution with respect to age groups.

	Frequency (adjusted residual, % within serotype, % within age group)													
Serotype^c^	<5 years				5–59 years				≥60 years				Total^a^	
1	3	(0.1,	33.3,	6.4)	2	(−1.5,	22.2,	2.8)	4	(1.7,	44.4,	12.1)	9	(6.0)
3	0	(−0.7,	0.0,	0.0)	0	(−0.9,	0.0,	0.0)	1	(1.9,	100.0,	3.0)	1	(0.7)
14	3	(1.4,	60.0,	6.4)	0	(−2.1,	0.0,	0.0)	2	(1.0,	40.0,	6.1)	5	(3.3)
34	2	(2.1,	100.0,	4.3)	0	(−1.3,	0.0,	0.0)	0	(−0.8,	0.0,	0.0)	2	(1.3)
10A	0	(−0.7,	0.0,	0.0)	1	(1.1,	100.0,	1.4)	0	(−0.5,	0.0,	0.0)	1	(0.7)
11A/11D	0	(−1.2,	0.0,	0.0)	2	(0.7,	66.7,	2.8)	1	(0.5,	33.3,	3.0)	3	(2.0)
12F/12A	1	(0.1,	33.3,	2.1)	0	(−1.6,	0.0,	0.0)	2	(1.9,	66.7,	6.1)	3	(2.0)
15A	0	(−0.7,	0.0,	0.0)	1	(1.1,	100.0,	1.4)	0	(−0.5,	0.0,	0.0)	1	(0.7)
15B/15C	0	(−0.7,	0.0,	0.0)	0	(−0.9,	0.0,	0.0)	1	(1.9,	100.0,	3.0)	1	(0.7)
16F	0	(−1.0,	0.0,	0.0)	1	(0.1,	50.0,	1.4)	1	(1.0,	50.0,	3.0)	2	(1.3)
18C	1	(0.6,	50.0,	2.1)	1	(0.1,	50.0,	1.4)	0	(−0.8,	0.0,	0.0)	2	(1.3)
19A	1	(−1.2,	12.5,	2.1)	6	(1.6,	75.0,	8.5)	1	(−0.7,	12.5,	3.0)	8	(5.3)
19F	22	(1.7,	39.3,	46.8)	26	(−0.1,	46.4,	36.6)	8	(−1.7,	14.3,	24.2)	56	(37.1)
23F	3	(−1.1,	18.8,	6.4)	12	(2.4,	75.0,	16.9)	1	(−1.6,	6.3,	3.0)	16	(10.6)
35B	2	(0.8,	50.0,	4.3)	2	(0.1,	50.0,	2.8)	0	(−1.1,	0.0,	0.0)	4	(2.6)
35F/47F	1	(0.1,	33.3,	2.1)	1	(−0.5,	33.3,	1.4)	1	(0.5,	33.3,	3.0)	3	(2.0)
6A/6B	1	(−1.3,	11.1,	2.1)	6	(1.2,	66.7,	8.5)	2	(0.0,	22.2,	6.1)	9	(6.0)
7C/7B/40	0	(−0.7,	0.0,	0.0)	1	(1.1,	100.0,	1.4)	0	(−0.5,	0.0,	0.0)	1	(0.7)
7F/7A	0	(−1.2,	0.0,	0.0)	1	(−0.5,	33.3,	1.4)	2	(1.9,	66.7,	6.1)	3	(2.0)
9V/9A	0	(−0.7,	0.0,	0.0)	0	(−0.9,	0.0,	0.0)	1	(1.9,	100.0,	3.0)	1	(0.7)
ND	7	(0.4,	35.0,	14.9)	8	(−0.7,	40.0,	11.3)	5	(0.4,	25.0,	15.2)	20	(13.2)
Total^b^	47 (31.1)				71 (47.0)				33 (21.9)				151	

a Frequency (total % within serotype).

b Frequency (total % within age groups).

c Fisher's exact test, p = 0.03.

Abbreviation: ND, not-detected.

## Discussion

The increase in reports of pneumococcal infections associated with antibiotic resistant strains has prompted for better disease-control and prevention strategies. The in-demand information on local distribution of pneumococci is still very much insufficient. A few studies conducted previously have reported different findings, probably due to the study designs and different geographical distributions (and hence the populations involved) in the studies [7]–[9], [18]–[21]. To promote surveillance and continue monitoring of pneumococcal infections in Malaysia, this study was carried out to investigate the distribution of serotype specific strains in relation to various aspects of pneumococcal diseases and to evaluate the efficacy of PCV7 among local community.

Although current study represents a single centre-based investigation in Malaysia, the incidences of penicillin nonsusceptible strains were remarkably high. Comparing study outcomes reported by other researchers are not always feasible due to the variations in study design. Moreover, the available epidemiological data throughout the years is extremely scarce. To evaluate the trend in penicillin resistance, studies reported by Rohani *et al.* [22] and Nasir *et al.* [23] provided relatively good representation of local epidemiological data covering patients of all age groups and isolates from various clinical sites. Penicillin nonsusceptible strains had increased substantially from 10.9% [22] to 31.0% [23] within four years time and further increased to 50.3% based on current study. Additionally, resistant strains has also increased from 5.5% [22] to 20.0% [23] and slightly to 21.2% based on current study. Our study clearly addresses the overwhelming penicillin resistance among the local isolates of *S.pneumoniae* in recent years. Such drastic increment especially the nonsusceptible strains could mean further reduction in treatment outcomes for patients infected with pneumococcal diseases.

Serotype 19F and 23F were the predominant serotypes among local populations and this is in agreement with a previous study by Cheong *et al.* [21]. In addition, the pool of serotypes has become smaller towards higher penicillin resistance level. From the data, serotype 19F, 23F, and 6A/6B were present persistently in all three susceptibility groups and this suggests that tendency in developing penicillin resistance might be serotype-specific. On the other hand, serotype 34 and 9V/9A were present neither in S_p_ nor I_p_ but only in R_p_ group.

As age of patients is one of the important factors underlying pneumococcal diseases, the relationship between age and serotypes, clinical sites, and penicillin susceptibility were investigated in this study. Respiratory tract remains as the most important clinical site for pneumococcal infections where large numbers of isolates were recovered from NP, sputum, and tracheal secretions. Serotype 19F was essentially the most abundant serotype particularly among younger age patients. Children <5 years old and the elderly adults are the major age groups frequently infected with pneumococcal diseases. It was noted that serotype 19A has a great potential in causing invasive pneumococcal diseases.

To evaluate the efficacies of PCV7 among the Malaysian population, serotypes covered under the vaccine were analyzed. This PCV7 covers serotype 4, 6B, 9V, 14, 18C, 19F, and 23F and also provides protection against those serotypes of the same serogroup as the formulated types. It was determined that 87.5% R_p_ (28/32), 79.5% I_p_ (35/44), and 45.3% S_p_ (34/75) are covered by the current PCV7. This vaccine appears to be able to confer protection against 64.2% of all incidences (97/151). Therefore, effectiveness of PCV7 to our community would be encouraging. Towards reducing the morbidity and mortality associated with pneumococcal infections, it is highly recommended that PCV7 to be incorporated in the regular vaccination scheme for children in Malaysia. This will help to reduce the incidence directly in those young children as well as indirectly as a result of herd community effect. Children with certain chronic diseases or preexisting medical conditions which put them at high risk of developing IPD should be considered for the vaccination. This includes sickle cell disease, asplenia, immunosuppression, renal disease, cerebrospinal fluid leaks, HIV infection, and others. Nonetheless, it is difficult to predict the shift in serotype prevalence in the future. Thus, continuing surveillance of pneumococcal carriage and diseases-related cases will help to characterize the effectiveness of PCV7 and monitoring of disease burden due to non-vaccine serotypes are certainly needed. This information will also provide the local regulatory body to recommend suitable vaccines to be used.

It is particularly interesting as how these serotypes are related to penicillin-resistance and the associated intracellular alterations that lead to the changes against penicillin stress. Many other minor and non-prevelant serotypes have also been detected among all three groups. This may be due to variations in the geographical area, disease and age groups of the patients. However, the high prevalence of penicillin resistance in this study suggests the rapid spread of resistant clones among pneumococci strains in this region.

Since pneumococci has the ability to switch serotypes by horizontal transfer recombination and other genetic events, it is important to monitor the frequency of the serotype exchanges in order to predict long term efficacy of new vaccines. Therefore continuous monitoring of antimicrobial resistance and serotype distribution of *S. pneumoniae* is important for diseases management and various disease-control policies among the Malaysian population. The current study supports the need for a long term surveillance program and also vaccination as measures for prevention and control of pneumococcal infections. We hope that current study will contribute to the regional body of knowledge on *S. pneumoniae* serotypes distribution in Malaysia, such as the Asian Strategic Alliance for Pneumococcal Diseases Prevention (ASAP) in align to their mission on control and contain pneumococcal diseases in Asian countries.

In conclusion, the rising threats of penicillin resistance among the Malaysian pneumococcal isolates were clearly described in this study. To our knowledge, such high incidences of penicillin nonsusceptible *S.pneumoniae* among Malaysian populations is first reported in this study. Due to the dominant role associated with serotype 19F against various aspects of pneumococcal diseases, reduced penicillin susceptibility among serotype 19F is of critical concern. Aggravation might continue if no effective measures regulating improper use of antibiotics is defined. These serotypes as well as other minor vaccine and vaccine-related types have been included in the 7-valent conjugate vaccine. Therefore, the efficacy of this vaccine should be adequate in our population. However, it is not possible to predict serotypes that might become predominant in the future.

## Supporting Information

Table S1 The concentrations of primers used in seven multiplex PCR reactions.
(DOC)
