# Supplementary material for: Capsular Serotype and Antibiotic Resistance of Streptococcus pneumoniae Isolates in Malaysia
Source: PLoS One. 2011 May 16;6(5):e19547. doi: 10.1371/journal.pone.0019547 (PMC3095606; doi:10.1371/journal.pone.0019547)
Supplement: Table S1 — The concentrations of primers used in seven multiplex PCR reactions. (DOC) [file pone.0019547.s001.doc]

| **Reaction** | **Primers** | **Primer concentration (µM)** |
| --- | --- | --- |
| 1 | 3-f, 3-r | 1.5 |
|  | 6A/B-f (biotin), 6A/B-r | 0.5 |
|  | 19A-f, 19A-r | 1.0 |
|  | 22F-f, 22-r | 1.5 |
|  |  |  |
| 2 | 4-f, 4-r | 1.5 |
|  | 9V-f, 9V-r | 1.5 |
|  | 12F-f, 12F-r | 1.5 |
|  | 14-f, 14-r | 1.0 |
|  |  |  |
| 3 | 7F-f, 7F-r | 2.0 |
|  | 11A-f, 11A-r | 1.0 |
|  | 23F-f, 23F-r | 1.5 |
|  | 33F-f, 33F-r | 1.0 |
|  |  |  |
| 4 | 16F-f, 16F-r | 2.0 |
|  | 19F-f, 19F-r | 1.5 |
|  | 35B-f, 35B-r | 1.0 |
|  | Sg18-f, sg18-r | 1.25 |
|  |  |  |
| 5 | 8-f, 8-r | 1.5 |
|  | 15B/C-f, 15B/C-r | 1.5 |
|  | 31-f, 31-r | 2.0 |
|  | 38-f, 38-r | 1.5 |
|  |  |  |
| 6 | 1-f, 1-r | 1.5 |
|  | 10A-f, 10-r | 1.5 |
|  | 34-f, 34-r | 1.5 |
|  | 35F-f, 35F-r | 1.5 |
|  |  |  |
| 7 | 7C-f, 20-r | 1.5 |
|  | 15A-f, 15A-r | 1.5 |
|  | 17F-f, 17F-r | 1.5 |
|  | 20-f, 20-r | 1.5 |

* *cpsA*-f and *cpsA*-r, the primers targeting the common cps operon region were included in each reaction at 0.5µM each.
